# Depressive Symptoms and Complications Early after Acute Myocardial Infarction: Gender Differences

**DOI:** 10.2174/1874434601812010205

**Published:** 2018-09-17

**Authors:** Mohannad Eid AbuRuz, Ghadeer Al-Dweik

**Affiliations:** College of Nursing, Applied Science Private University, Amman, Jordan

**Keywords:** Depressive symptoms, Acute myocardial infarction, Complication, Developing countries, Coronary Heart Disease (CHD), CVD

## Abstract

**Background:**

Cardiovascular disease is the first leading cause of death worldwide. Coronary heart disease is the most common manifestation of cardiovascular disease. Acute myocardial infarction is the primary manifestation of coronary heart disease. Depression is a common and predicted complication after acute myocardial infarction. Limited studies evaluated gender differences in depressive symptoms after acute myocardial infarction especially in developing countries.

**Objective:**

The study aimed to determine whether there was a difference in depression levels and rate of complications based on gender early after acute myocardial infarction.

**Method:**

This was a prospective comparative study on 230 patients (150 men and 80 women) with a confirmed diagnosis of acute myocardial infarction. All participants signed an informed consent, filled sociodemographic and clinical questionnaire and the Depression Subscale of the Hospital Anxiety and Depression Scale. Clinical data were abstracted from the participants’ medical record after discharge.

**Results:**

Eighty-six participants (37.4%), 54 men and 32 women, developed 1 or more complications during hospitalization. Female patients were more depressed (14.4±3.5 *vs.* 8.3 ± 2.6) and developed more complications (1.9 ± 0.9 *vs.* 0.8 ± 0.5) than male patients did. Depressive symptoms increased the occurrence of complication by 40% and 33% for female and male patients respectively after controlling for sociodemographic and clinical variables.

**Conclusion:**

Depressive symptoms independently predicted complications after acute myocardial infarction in both men and women. The inclusion of depression assessment tools in acute myocardial infarction treatment protocols is highly recommended.

## 1 INTRODUCTION

Cardiovascular Disease (CVD) is the first leading cause of death in many countries [1, 2]. The most common manifestation of CVD is the Coronary Heart Disease (CHD) [3]. The prevalence of CHD in US was approximately 16 million [1]. It affected approximately 6.6 million US women annually and remained the leading cause of morbidity and mortality threat in women. Of these, 2.7 million had a history of acute myocardial infarction (AMI) among which >53,000 died of this AMI, and an estimated 262,000 women were hospitalized for an acute coronary syndrome (AMI and unstable angina) [1].

In Europe, about 55% of all female deaths are caused by CVD, especially CHD, compared with 44% of all male deaths [4]. The mortality of CHD will increase by 174% in men and 146% in women in the Middle East countries [5]. In Jordan, a Middle Eastern developing country, according to the latest WHO report, CHD deaths reached nearly 19% of total deaths. The death rate per 100,000 due to CHD is 131of population, ending to make Jordan to be ranked number 46 in the world [2].

Acute myocardial infarction is the primary manifestation of CHD. Depression is associated with negative short and long-term outcomes among patients with AMI [6, 7]. Depression is common and persistent in AMI survivors [7, 8]. Growing evidence supports that post-AMI depression is an independent risk factor for future cardiac events and mortality [6, 7, 9-12]. Furthermore, it has been shown that depression predicted complications after AMI [7, 9, 13-15]. Most of these complications occur during the early phases of the event and might begin as early as the first 20 minutes [16-19].

In previous studies, different complications have been reported in patients who developed AMI including arrhythmias (*i.e.* ventricular tachycardia, ventricular fibrillation), inflammation (early pericarditis and post-AMI syndrome), as well as left ventricular mural thrombus. In addition, acute recurrent ischemia, re-infarction, cardiogenic shock, and in-hospital death were also reported [7, 15-19].

Depression is a prevalent phenomenon across the world [20-22]. With few exceptions, females have a higher incidence and level of depressive symptoms compared to males [10, 20-25]. For instance, in a meta-analysis of 16 prospective studies about sex differences in depression post AMI, 14 studies with 7202 participants out of 10175 reported that females had higher levels of depressive symptoms compared to males [20]. Usually, the rate of depressive symptoms is higher post AMI compared to the general population [7, 20].

Depression is the most common mental illness in females who have twice the risk of major depressive disorder as compared with males [26-29]. Population studies from Canada [30], Germany [31], and Switzerland [32] all reported that females are at least twice as likely as males to suffer from major depressive disorder. A prospective study conducted in US to examine the extent to which depressive symptoms accounted for the higher rates of post-AMI adverse outcomes in women concluded that women have a higher prevalence of depressive symptoms after AMI compared with men [33]. Data from large epidemiological studies revealed that males and females often report distinct differences in the self-reported somatic depressive symptoms of depression [34, 35]. Somatic depressive symptoms include sleep disturbance, appetite disturbance, and fatigue for at least two weeks [34, 35].

In a scientific statement from the American Heart Association about depression as a risk factor for poor prognosis among patients with acute coronary syndrome, 24 out of 53 studies included AMI patients [36]. Nearly all these 24 studies used self-reported questionnaires to assess depression. Among these 24 studies; 21 studies found an association between depression and all-cause mortality. The remaining three studies did not find a significant association [36]. Moreover, in this statement 11 studies checked the effect of depression on cardiac mortality after AMI. Among these studies; 8 studies reported a significant relationship between depression and cardiac mortality. However, the remaining three studies did not report any significant relationship [36]. Based on that the American Heart Association supported the evaluation of depression as a risk factor for complications and mortality after acute coronary syndrome [36].

Unfortunately, in developing countries, medical research has historically neglected the health needs of women, apart from reproductive concerns. Multiple studies have shown that women with acute coronary syndromes are less likely to be treated with guideline-directed medical therapies [37, 38]. Improving AMI morbidity and mortality and closing the knowledge gaps on AMI clinical presentations considering the gender differences and treatments for women are public health priorities. Limited studies have specifically evaluated gender differences in specific depressive symptoms reported after AMI event especially in developing countries. There is a lack of research regarding gender differences among Jordanian patients with AMI. Therefore, the purpose of this study was to determine whether there was a difference in depression levels and rate of complications based on gender early after AMI.

### 1.1 Research Hypotheses

1. Female patients will have higher depression scores than male patients.
2. Female patients will have a higher complication rate than male patients.
3. Depression scores will be an independent predictor for complications (for men and women) after controlling for sociodemographic and clinical variables.

## 2 MATERIAL AND METHODS

### 2.1 Research design, Sample, and Setting

To meet the objective of this study, a prospective comparative design was used. The study was conducted at 3 private hospitals in Amman, Jordan. The inclusion criteria were (1) a confirmed diagnosis of AMI by elevated cardiac enzymes, standard electrocardiogram (ECG) changes, and/or chest pain; (2) 18 years and older; (3) no chest pain and hemodynamic stable at the time of interview; (4) no cognitive impairment affecting the ability to answer questionnaires; and (5) no previously diagnosed psychiatric disorder. A power analysis based on Cohen power table was conducted [39] to ensure that the sample size was sufficient to detect statistical significance based on the following criteria; a) medium effect size, b) a power of 0.80, c) a type I error of 0.05, and d) the statistical tests were mean difference for hypotheses 1 and 2, and regression with 7 independent variables for hypothesis 3. Based on these assumptions, 64 participants were needed for hypotheses 1 and 2, whereas 106 participants were needed for hypothesis 3. Therefore, it was determined that the sample size of 230 was adequate.

### 2.2 Ethical Considerations

This study was reviewed and approved by the institutional review board (IRB) committee at the Applied Science Private University, Amman, Jordan (faculty 005). Thereafter, the principal investigator presented the study proposal and submitted the IRB approval letter to the medical and nursing directors of the selected hospitals. These institutions acknowledged the IRB approval from the Applied Science Private University. Therefore, permissions to carry out the study within these hospitals were issued to the principal investigators by the medical directors.

### 2.3 Procedure

Experienced cardiovascular research assistants met with the participants and explained the study objectives. Participants were assured that participation was voluntary and they could withdraw from the study at any time. Then, they signed an informed consent if they agreed to participate. Research assistants collected data from each participant within 72 hours (mean [SD], 38 hours [19]) of admission to the hospital. The following were completed during the interview; sociodemographic and clinical questionnaire and the Depression Subscale of the Hospital Anxiety and Depression Scale (HADS). In addition, clinical data were abstracted from the participants’ medical records after discharge.

### 2.4 Measurement of Variables

#### 2.4.1 Sociodemographic and Clinical Characteristics

Data were obtained by research assistants either by face to face interview or by reviewing medical records. Sociodemographic and clinical data collected were: age, gender, marital status, admission vital signs, smoking history, medications history, and medical history of (hypertension, diabetes, previous myocardial infarction, and previous angina), surgical history/intervention of (coronary artery bypass graft, percutaneous transluminal intervention, and stent use). In addition, we measured the Intensive Care Unit (ICU) and hospital Lengths Of Stay (LOSs), Left Ventricular Ejection Fraction (LVEF) from the medical records.

#### 2.4.2 Depression

The depression subscale of the HADS was used to measure depression in this study. This subscale is translated into Arabic, short, easy to use and interpret, valid, and reliable [40-44]. The Cronbach’s for the Arabic version of this subscale was 87. The sensitivity and specificity were at 79% and 87%, respectively [41, 43, 44]. The HADS consists of 7-items in which the participants rated each item on a scale of 0 to 3, with 3 indicating higher symptom frequency and severity. The items were summed to a total score that ranged from 0 to 21, with higher scores indicating higher levels of depressive symptoms. The scores were categorized as follows: 0 to 7, normal; 8 to 10, mild; 11 to 14, moderate; and 15 to 21, severe depression [40].

#### 2.4.3 In-hospital Complications

Complications were defined as in previous studies [3, 7, 17, 18, 45] as the occurrence of any of the following during hospitalization: (a) reinfarction evidenced by elevated cardiac enzymes and standard ECG changes; (b) supraventricular tachyarrhythmia with hemodynamic instability, (c) acute recurrent ischemia evidenced by new onset of chest pain, with ECG changes or hemodynamic instability; (d) sustained ventricular tachycardia (≥30 seconds) or any ventricular tachycardia requiring cardioversion and/or pharmacological intervention; (e) ventricular fibrillation; (f) cardiogenic shock; (g) acute pulmonary edema; or (h) in-hospital death.

### 2.5 Data Analysis

Data were analyzed using SPSS software version 21.0 (SPSS Inc, Chicago, Illinois). *P*-Value less than .05 was set a priori. Sociodemographic and clinical characteristics of the sample based on gender were described using frequencies and percentages or mean (SD). Independent-sample t-test was used to test the first 2 hypotheses of the study. Correlations between depression scores for both genders and the total number of complications, ICU LOS, and hospital LOS were examined using the Pearson r correlation coefficient. Predictors of complication for both genders (hypothesis 3) were tested using logistic regression.

### 3 RESULTS

230 patients participated in this study, 150 men and 80 women. Table **1** presents the sociodemographic and clinical characteristics of the sample by gender. Three-quarters of the sample was hypertensive, more than half of the sample had previous AMI, and the majority had a history of angina. Male patients more often had diabetes (*X*^2^= 8.01, P < .01), had a higher prevalence of previous angina (*X*^2^= 11.02, P < .01), and had coronary artery bypass graft surgery more commonly (*X*^2^= 8.87, P < .01) than female patients did. However, female patients have significantly lower levels of LVEF compared to male patients.

Eighty-six participants (37.4%), 54 men and 32 women, developed 1 or more complications during hospitalization Table **2**. Female patients were more depressed and developed complications more than male patients did Table **3**. Female patients were moderately depressed based on the operational definition of HADS with a mean (SD) of 14.4 (3.5). Male patients were mildly depressed based on a mean (SD) for the HADS of 8.3 (2.6).

The correlation between depression scores for males and females and the total number of complications, ICU LOS, and hospital LOS are presented in Table **4**. The only significant correlation was between the number of complications with the depression scores for female patients (r = 0.21, P < .05).

Two separate logistic regression equations were used to predict the occurrence of complications using the same predictors entered for male and female patients. History of hypertension and depression scores were independent predictors of complication for male patients Table **5**, whereas depression scores were the only predictor for complication of female patients Table **6**. Depressive symptoms increased the risk for development of complication for men by 33% and for women by 40%.

## 4 DISCUSSION

This was the first study specifically designed to check the differences in depression levels and complication rate after AMI between males and females in a developing country. The major findings of this study were that female patients had higher levels of depressive symptoms and complications compared to male patients. In addition, depressive symptoms were independent predictor of these complications for male and females at the same time.

The results of this study showed that females have more depressive symptoms than males. This result is consistent with previous studies [15, 20, 25, 26, 46-48] conducted on the same topic. Different explanations were proposed for this result. *First:* Previous research showed that poor LVEF was associated with depression in patients with coronary artery disease [20, 49], heart failure [20, 50], and post AMI [20, 51]. Moreover, low levels of LVEF were important predictors for poor quality of life in all domains for heart failure patients following AMI, including mental and physical components [52, 53]. In this study, female patients had significantly lower LVEF compared to males which might explain why they had higher levels of depressive symptoms. *Second:* Women may more often have preexisting depression prior to the AMI event compared to men, which might exaggerates the depressive symptoms after AMI [54]. *Third:* Recently, research starts to focus on the relationship between fetal exposure and the increased risk for depression in females, which still needs further studies [55]. *Fourth:* Previous research found that females had higher levels of fatigue after AMI event, and fatigue was positively correlated with depressive symptoms. Further research explaining why females have more depression after AMI is still warranted.

The results of this study also showed that depressive symptoms were independent predictors of in-hospital complication for both males and females. These results are consistent with previous studies about the relationship between depression and in-hospital complications [7, 15, 56, 57]. The most recent study [7] on 175 post AMI patients demonstrated that depressive symptoms were associated with increased risk of in-hospital complications including acute recurrent ischemia, re-infarction, sustained ventricular tachycardia, ventricular fibrillation; or in-hospital death. Kerrat *et al* (2010) [15] measured depression by BDI in 906 patients with AMI and unstable angina. Among them, 492 (58.4%) developed complications including ischemia, sudden cardiac arrest, arrhythmia, re-infarction, major bleed, and had a temporary transvenous pacemaker. The study showed that depressive symptoms were significantly associated with these complications. Moreover, ischemia was the most prevalent complication as in the current study.

Huffman *et al* (2008) [11] showed that clinical depression measured by interview was associated with cardiac complications in 129 AMI patients. These complications included ventricular arrhythmias, heart failure and recurrent AMI. These results are consistent with the results by dickens *et al.* [57] who found that clinical depression was an independent predictor of the occurrence of heart failure after AMI.

Contrary, Cherrington *et al* [58] checked the association between depressive symptoms and anxiety with in-hospital complications after AMI. They found that neither depressive symptoms nor anxiety was associated with in-hospital complications. A possible explanation might be the small sample size of the study; there were only 49 patients enrolled in this study.

Most of the previous studies about the difference between female and male patients focused on long term complications and mortality. Limited number of studies [3, 15, 23, 47] checked the differences between female and male patients in the development of *in-hospital* complications after AMI. In the current study, female patients developed more complications than male patients. This result is consistent with different previous studies about in-hospital complication.

In this study and Grewal *et al* study [15] described previously, female patients had higher levels of depressive symptoms than male patients. Since depression is strongly associated with complication, it is no wonder that female patients developed more complications than male patients. Moreover, female patients in the current study had lower levels of LVEF compared to male patients giving further support for these results. It has also been shown in another study about anxiety and complications after AMI in a developing country that female patients developed more complications compared to male patients [3]. Female patients were severely anxious, while male patients were mildly anxious which explain the results.

In a study of 11,896 Chinese patients recruited from 162 Chinese hospitals [47], the overall in-hospital mortality rate was higher in women compared to men, even after adjustment for sociodemographic and clinical characteristics. The same results were obtained from an Italian study [23] which enrolled 89,562 patients with a confirmed diagnosis of ST-elevation MI. In-hospital mortality was higher among female patients compared to male patients. However, there was an age-gender interaction with female gender being a significant predictor of increased mortality in patients aged ≥75 years.

Contrary, only one study [59] reported that male patients had more complications, namely (severe arrhythmias), than female patients. It is worthy to note that the sample of this study consisted of (9737) patients suffering an episode of chest pain aged between 25-74 years who were admitted to the CCU. Signs and symptoms of AMI were presented in 2500 male patients and 979 female patients. Male patients were taking antiarrhythmias more than female patients which might explain the results of the study. Antiarrhythmias medications usually have the development of new arrhythmias as one of their side effects.

## CONCLUSION AND IMPLICATION

This was the first study to check the effect of depressive symptoms and gender differences on in-hospital complication after AMI in a developing country. The results showed that depressive symptoms increased in-hospital complications after AMI. Female patients have more depressive symptoms than male patients did, which made them more susceptible to these complications. These results are approximately the same with the results obtained from developed countries indicating that depression is a global problem after AMI, especially for women. Previous studies demonstrated that effective treatment of depression reduced mortality in depressed post AMI patients. However, depression in this population cannot be treated unless it is firstly identified. Therefore, it is highly recommended to include depression assessment in AMI treatment protocols. Future studies to determine the physiological and psychological mechanisms to explain this difference between female and male patients are still needed.

## Figures and Tables

**Table 1:** Sociodemographic and clinical characteristics of the sample by gender.

Variable	Total sample (n=230)	Males (n=150)	Females (n = 80)
Age	66.6 ± 11.2	65.5 ± 11.1	67.5 ± 10.6
LVEF	30.8 ± 6.0	32.5 ± 8.2	29.8 ± 4.0*
History of hypertension	173 (75.2)	118 (78.7)	55 (68.8)
History of diabetes	89 (38.7)	68 (42.3)	21 (26.3)**
History of previous AMI	138 (60.0)	93 (62.0)	45 (56.3)
History of previous angina	190 (82.6)	133 (88.7)	57 (71.3)**
History of previous CABG	134 (58.3)	98 (65.3)	36 (45.0)**
History of stent use	101 (43.9)	71 (47.3)	30 (37.5)
Had any complication during hospitalization	86 (37.4)	54 (36.0)	32 (40.0)
Smoking Never smoked Current smoker Former smoker	71 (30.9) 57 (24.8) 102(44.3)	34 (22.7) 46 (30.7) 70 (76.7)	37 (46.3) 11(13.8) 32 (40.0)

LVEF: left ventricular ejection fraction, AMI: Acute myocardial infarction, CABG: coronary artery bypass graft, *significant at p <.05, **significant at p <.01

**Table 2:** Specific complications developed and their percentages.

Complication Developed	*Number of Patients (%)
Acute recurrent ischemia	52 (22.6)
Sustained ventricular tachycardia	12 (5.2)
Pulmonary edema	7 (3.0)
Re-infarction	7 (3.0)
Cardiogenic shock	6 (2.6)
In-hospital death.	6 (2.6)
Ventricular fibrillation	5 (2.2)
*More than one patient developed more than one complication	

**Table 3:** Comparison of depression level and complication based on gender.

Item	Gender	Means	SD	P Value
Depression level (HADS; depression subscale scores)	Female Male	14.4 8.3	3.5 2.6	<.01
Complications	Female Male	1.9 0.8	0.9 0.5	<.05

HADS: Hospital anxiety and Depression Scale.

**Table 4:** Correlation between depression scores based on gender with complications and LOS.

Variable	Complication	ICU LOS	Hospital LOS
depression Scores/ male patients	0.02 (NS)	0.03 (NS)	0.06 (NS)
depression Scores/ female patients	0.21*	0.02 (NS)	0.07 (NS)

LOS: Length of stay, ICU: Intensive Care Unit, *significant at P < .05, NS: Not significant.

**Table 5:** Predictors of complication for male patients.

Predictor	OR	95% confidence interval	B	Wald	P- value
Age	1.02	0.99-1.08	0.03	3.4	0.07
Previous AMI	0.74	0.35-1.55	-0.31	0.66	0.42
Diabetes	0.90	0.43-1.90	-0.11	0.08	0.78
Hypertension	2.53	1.07-6.43	0.93	3.77	***<*0.05**
Previous CABG	1.89	0.88-4.10	0.64	2.65	0.10
Depression scores	1.33	1.21-2.24	0.19	28.15	***< 0.001***

OR: Odds ratio, AMI: Acute Myocardial Infarction, CABG: Coronary Artery Bypass Graft Surgery

**Table 6:** Predictors of complication for female patients.

Predictor	OR	95% confidence interval	B	Wald	P- value
Age	1.00	0.96-1.05	0.001	0.002	0.98
Previous AMI	0.48	0.18-1.25	-0.74	2.27	0.13
Diabetes	0.75	0.26-2.13	-0.29	0.29	0.59
Hypertension	1.45	0.52-4.08	0.37	0.50	0.48
Previous CABG	0.94	0.36-2.44	-0.06	0.02	0.90
Depression scores	1.40	1.09-1.88	0.61	39.32	***<0.001***

OR: Odds ratio, AMI: Acute Myocardial Infarction, CABG: Coronary Artery Bypass Graft Surgery
